# Clinical Value of Core Length in Contemporary Multicore Prostate Biopsy

**DOI:** 10.1371/journal.pone.0123704

**Published:** 2015-04-14

**Authors:** Sangchul Lee, Seong Jin Jeong, Sung Il Hwang, Sung Kyu Hong, Hak Jong Lee, Seok Soo Byun, Gheeyoung Choe, Sang Eun Lee

**Affiliations:** 1 Department of Urology, Seoul National University Bundang Hospital, Seongnam, Korea; 2 Department of Radiology, Seoul National University Bundang Hospital, Seongnam, Korea; 3 Department of Pathology, Seoul National University Bundang Hospital, Seongnam, Korea; University of Colorado Denver, UNITED STATES

## Abstract

**Objectives:**

There is little data about the clinical value of core length for prostate biopsy (PBx). We investigated the clinical values of various clinicopathological biopsy-related parameters, including core length, in the contemporary multi-core PBx.

**Patients and Methods:**

Medical records of 5,243 consecutive patients who received PBx at our institution were reviewed. Among them, 3,479 patients with prostate-specific antigen (PSA) ≤10ng/ml level who received transrectal ultrasound (TRUS)-guided multi (≥12)-core PBx at our institution were analyzed for prostate cancer (PCa). Gleason score upgrading (GSU) was analyzed in 339 patients who were diagnosed with low-risk PCa and received radical prostatectomy. Multivariate logistic regression analyses for PCa detection and prediction of GSU were performed.

**Results:**

The mean age and PSA of the entire cohort were 63.5 years and 5.4ng/ml, respectively. The overall cancer detection rate was 28.5%. There was no statistical difference in core length between patients diagnosed with PCa and those without PCa (16.1 ± 1.8 vs 16.1 ± 1.9mm, *P* = 0.945). The core length was also not significantly different (16.4 ± 1.7 vs 16.4 ± 1.6mm, *P* = 0.889) between the GSU group and non-GSU group. Multivariate logistic regression analyses demonstrated that the core length of PBx did not affect PCa detection in TRUS-guided multi-core PBx (*P* = 0.923) and was not prognostic for GSU in patients with low-risk PCa (*P* = 0.356).

**Conclusions:**

In patients undergoing contemporary multi-core PBx, core length may not have significant impact on PCa detection and also GSU following radical prostatectomy among low-risk PCa group.

## Introduction

Random prostate biopsy (PBx) is the most important diagnostic tool for detecting prostate cancer (PCa) and provides a cancer detection rate (DR) between 20% and 40% in the initial biopsy setting [1]. The concept of target biopsy may improve the low diagnostic yield of random PBx for the detection of cancer. Target biopsy involves sampling the prostate using imaging modalities, such as real-time elastography or multi-parametric magnetic resonance imaging (MRI). Another possible strategy to increase the cancer DR would be to obtain more prostate tissue through methods, such as extended PBx or saturation PBx [2]. Several studies report that adding laterally directed cores to the traditional sextant PBx scheme significantly increases the cancer DR without significantly increasing PBx-related morbidity [3, 4]. A recent systematic review and meta-analysis reports that the saturation PBx scheme demonstrates a significant advantage in PCa detection [5]. Another strategy to increase the cancer DR might be to obtain longer cores with PBx. The length of prostate cores sampled at biopsy is considered to be a quality indicator of PBx [6, 7]. However, there is a paucity of data in the literature regarding the clinical value of core length in random PBx for detecting cancer.

The number of men diagnosed with low-risk PCa has recently increased due to the introduction of prostate-specific antigen (PSA) screening [8]. In patients with low-risk PCa, radical prostatectomy has failed to improve cancer-specific survival compared to watchful waiting in randomized studies [9, 10]. Thus, active surveillance (AS) is the main treatment option for patients with low-risk PCa. However, about a third of patients who are initially identified as low risk for PCa development based on PBx experience Gleason score upgrading (GSU) following immediate radical prostatectomy [11, 12]. GSU increases the risk of biochemical recurrence and reduces cancer-specific survival [13]. Indeed, GSU is a source of psychological stress for both clinicians and patients who are under AS. Thus, the prediction of GSU in patients diagnosed with low-risk PCa would be helpful for selecting candidates for AS. A number of clinicopathological parameters based on PBx have been introduced as prognostic factors for predicting the likelihood of GSU after histopathological diagnosis of the whole prostate in patients with PCa [14]. However, there is no report regarding the prognostic value of core length in PBX for the prediction of GSU in patients with low-risk PCa.

We investigated the clinical values of various clinicopathological biopsy-related parameters, including core length, in the contemporary multi (≥12)-core PBx.

## Materials and Methods

### Ethics Statement

The Institutional Review Board of Seoul National University Bundang Hospital (Seongnam, Republic of Korea) approved this study (approval number: B-1402/240-103). The need for informed consent from patients was waived by the Institutional Review Board because this study was a retrospective analysis. The records and information of patients were anonymized and de-identified prior to analysis.

### Patient Population

The medical records of 5,243 consecutive patients who received PBx at our institution between November 2003 and June 2012 were reviewed. Among these patients, 3479 patients with PSA values ≤10 ng/ml who received transrectal ultrasound (TRUS)-guided multi (≥12)-core PBx in the initial setting were finally included for the analysis of PCa detection. We excluded patients who were diagnosed with other cancers, such as transitional cell carcinoma, and those for whom data were missing.

Among patients diagnosed with PCa by TRUS-guided contemporary multi (≥12)-core PBx at our institution, 339 who were classified as having low-risk PCa [PSA ≤10 ng/ml, biopsy Gleason sum ≤6, clinical stage ≤T2a] and received radical prostatectomy after January 2007 were included for the analysis of GSU prediction.

### TRUS-guided PBx and Pathologic Diagnosis

TRUS-guided PBx was performed by two experienced radiologists. The 12 extended systematic biopsies usually included six cores of lateral peripheral zone biopsies and six cores of medial peripheral zone biopsies. If more than 12 core biopsies were performed, the transitional zone or suspicious lesions observed on TRUS were also included. After applying a periprostatic nerve block with 10 cc of lidocaine in a 22-gauge needle, TRUS-guided biopsy was performed with an automatic spring-loaded device and 20-cm, 18-gauge needle (TSK ACECUT; TSK Laboratory, Japan).

Pathological diagnoses of specimens retrieved with TRUS-guided PBx or by radical prostatectomy were consistently done by one experienced pathologist. The percentage of positive cores (%) was defined as the number of positive cores divided by the total number of cores obtained ×100; the maximum percentage of tumor length in a positive core (%) was defined as the maximum length of cancer on the most involved core divided by the length of the most involved core ×100. The percentage of total tumor length in cores (%) was defined as the total cancer length divided by the total length of cores obtained ×100.

### Examination of Clinicopathologic Factors

We evaluated the clinicopathological factors, including age, body mass index (BMI), PSA, prostate volume, number of biopsy cores taken, clinical stage, core length, abnormal finding with hard nodule on digital rectal examination, abnormal TRUS finding with suspicious hypoechoic lesion by standard grey-scale TRUS technology, percentage of positive cores, maximum percentage of tumor length in a positive core, and percentage of total tumor length in cores.

### Statistical Analysis

The primary clinicopathological outcomes were compared using Student’s t-test and chi-square test for the entire cohort and low-risk PCa cohort, respectively. Univariate and multivariate logistic regression analyses for PCa detection and GSU prediction were performed. All *P* values were two-sided, and values <0.05 were considered to be statistically significant. All data analyses were performed using the Statistical Package for the Social Sciences 18.0 software (SPSS Inc., Chicago, IL, USA).

## Results

Among 5,243 patients, we excluded patients for whom PSA >10 ng/ml, those who had repeat PBx, those for whom the number of PBx cores <12, those who had other cancers, such as transitional cell carcinoma, on PBx pathology, and those for whom data were missing. Finally, clinicopathogical data of 3,479 patients were analyzed. The mean age and PSA of the final cohort were 63.5 ± 9.1 years and 5.4 ± 2.0 ng/ml, respectively. The overall cancer DR was 28.5%. The mean age and PSA at the time of the PBx in the cancer group were higher than those of the non-cancer group (66.5 ± 7.4 vs. 62.3 ± 9.4 years, *P* < 0.001 and 5.8 ± 2.1 vs. 5.2 ± 2.0 ng/ml, *P* < 0.001, respectively). The cancer group showed a smaller prostate volume (38.0 ± 15.4 vs. 44.6 ± 19.0 ml, *P* < 0.001), higher proportion of abnormal findings with hard nodules on digital rectal examination (18.8 vs. 10.6%, *P* < 0.001), and higher proportion of TRUS findings with suspicious hypoechoic lesions (22.7 vs. 13.1%, *P* < 0.001). However, there were no significant differences between the two groups in terms of BMI, number of biopsy cores taken, mean total core length, and mean core length according to PBx sites (apex, mid-gland, base) (Table 1).

**Table 1:** Comparison of clinicopathological features among men who received contemporary multicore prostate biopsy and had PSA levels ≤ 10 ng/ml.

	Entire cohort	Cancer	No cancer	*P* value
Number of patients (%)	3479	992 (28.5)	2487 (71.5)	
Age (years)	63.5 ± 9.1	66.5 ± 7.4	62.3 ± 9.4	< 0.001
BMI (kg/m^2^)	24.4 ± 2.7	24.4 ± 2.8	24.4 ± 2.7	0.382
PSA (ng/ml)	5.4 ± 2.0	5.8 ± 2.1	5.2 ± 2.0	< 0.001
Prostate volume (ml)	42.7 ± 18.3	38.0 ± 15.4	44.6 ± 19.0	< 0.001
Number of biopsy cores taken	12.5 ± 1.1	12.6 ± 1.0	12.5 ± 1.1	0.622
Core length (mm)	16.1 ± 1.9	16.1 ± 1.8	16.1 ± 1.9	0.945
Apex core length (mm)	16.3 ± 2.0	16.3 ± 1.9	16.4 ± 2.1	0.806
Mid-gland core length (mm)	16.4 ± 2.0	16.3 ± 2.2	16.4 ± 2.2	0.452
Base core length (mm)	15.7 ± 2.0	15.8 ± 2.0	15.7 ± 2.0	0.536
Abnormal DRE (%)	12.9	18.8	10.6	< 0.001
Suspicious lesion on TRUS (%)	15.8	22.7	13.1	< 0.001

Abbreviations: BMI, body mass index; PSA, prostate-specific antigen; DRE, digital rectal examination; TRUS, transrectal ultrasound

Data are presented as means ± standard deviations.

Table 2 shows the univariate and multivariate analyses of clinicopathological factors in the prediction of cancer detection. Age (*P* < 0.001, OR = 1.076, 95% CI: 1.065–1.087), PSA (*P* < 0.001, OR = 1.213, 95% CI: 1.165–1.262), prostate volume (*P* < 0.001, OR = 0.959, 95% CI: 0.953–0.965), abnormal findings with hard nodules on digital rectal examination (*P* < 0.001, OR = 1.447, 95% CI: 1.158–1.810), and abnormal TRUS findings with suspicious hypoechoic lesions (*P* < 0.001, OR = 1.627, 95% CI: 1.311–2.019) were significantly associated with cancer detection on PBx in multivariate analysis (Table 2). However, the number of biopsy cores taken and core length were not predictive factors by multivariable analysis (*P* > 0.05).

**Table 2:** Univariate and multivariate analyses of clinicopathological factors and detection of prostate cancer via prostate biopsy.

	Univariate		Multivariate	
	OR (95% CI)	*P* value	OR (95% CI)	*P* value
Age (years)	1.060 (1.050–1.070)	< 0.001	1.076 (1.065–1.087)	< 0.001
PSA (ng/ml)	1.136 (1.096–1.178)	< 0.001	1.213 (1.165–1.262)	< 0.001
Prostate volume (ml)	0.976 (0.971–0.981)	< 0.001	0.959 (0.953–0.965)	< 0.001
Number of biopsy cores taken	1.017 (0.950–1.089)	0.622	1.083 (0.996–1.179)	0.063
Core length (mm)	0.999 (0.960–1.039)	0.945	1.002 (0.961–1.045)	0.923
Abnormal DRE	1.951 (1.591–2.394)	< 0.001	1.447 (1.158–1.810)	0.001
Suspicious lesion on TRUS	1.945 (1.610–2.349)	< 0.001	1.627 (1.311–2.019)	< 0.001

Abbreviations: OR, odds ratio; CI, confidence interval; PSA, prostate-specific antigen;

DRE, digital rectal examination; TRUS, transrectal ultrasound

Data from 339 patients diagnosed with low-risk PCa by TRUS-guided multi (≥12)-core PBx who received radical prostatectomy at our institution since January 2007 were evaluated for the prediction of GSU. The GSU group had a statistically smaller prostate volume (*P* < 0.001) and fewer biopsy cores taken (*P* = 0.008). The percentage of positive cores, maximum percentage of tumor length in a positive core, and percentage of total tumor length in cores in the GSU group were significantly higher than those of the non-GSU group (*P* < 0.001)(Table 3). However, there were no significant differences between the two groups in terms of age, BMI, pre-biopsy PSA, clinical stage, and core length (*P* > 0.05) (Table 3).

**Table 3:** Comparison of clinicopathological features among men diagnosed with low-risk prostate cancer according to Gleason score upgrading (GSU).

	Entire cohort	No GSU group	GSU group	*P* value
Number of patients (%)	339	102 (30.1)	237 (69.1)	
Age (years)	65.4 ± 6.8	64.6 ± 7.2	65.7 ± 6.6	0.168
BMI (kg/m^2^)	24.3 ± 2.7	24.3 ± 2.4	24.2 ± 2.8	0.877
PSA (ng/ml)	5.4 ± 1.9	5.1 ± 2.1	5.5 ± 1.9	0.104
Prostate volume (ml)	38.0 ± 14.3	43.1 ± 15.1	35.8 ± 13.3	< 0.001
Number of biopsy cores taken	12.4 ± 0.8	12.6 ± 0.9	12.3 ± 0.7	0.008
Core length (mm)	16.4 ± 1.7	16.4 ± 1.6	16.4 ± 1.7	0.889
Apex core length (mm)	16.7 ± 1.6	16.6 ± 1.8	16.7 ± 1.5	0.611
Mid-gland core length (mm)	16.7 ± 1.6	16.7 ± 1.8	16.7 ± 1.5	0.742
Base core length (mm)	16.1 ± 1.7	16.0 ± 1.8	16.2 ± 1.6	0.309
Percentage of positive cores (%)	19.8 ± 14.6	14.5 ± 11.1	22.1 ± 15.3	< 0.001
Maximum percentage of tumor length in a positive core (%)	21.1 ± 16.0	15.1 ± 11.6	23.6 ± 17.0	< 0.001
Percentage of total tumor length in cores (%)	3.2 ± 3.4	1.9 ± 2.3	3.7 ± 3.6	< 0.001
Clinical stage (%)				0.692
T1c	268 (79.1)	82 (80.4)	186 (78.5)	
T2a	71 (20.9)	20 (19.6)	51 (21.5)	
Very low-risk prostate cancer	137 (40.4)	64 (62.7)	73 (30.8)	< 0.001
Pathological T stage (%)				0.007
T2	307 (90.6)	99 (97.1)	208 (87.8)	
T3a	32 (9.4)	3 (2.9)	29 (12.2)	
T3b	0	0	0	
Positive surgical margin (%)	50 (14.7)	5 (4.9)	45 (19.0)	0.001

Abbreviations: GSU, Gleason score upgrading; BMI, body mass index; PSA, prostate-specific antigen

Data are presented as means ± standard deviations.

Table 4 shows the univariate and multivariate analyses of clinicopathological factors predicting GSU in men diagnosed with low-risk PCa. Multivariate logistic regression analysis also demonstrated age (*P* < 0.001, OR = 1.060, 95% CI: 1.019–1.102), PSA (*P* = 0.038, OR = 1.160, 95% CI: 1.008–1.336), prostate volume (*P* < 0.001, OR = 0.960, 95% CI: 0.941–0.981), and number of biopsy cores taken (*P* = 0.007, OR = 0.637, 95% CI: 0.458–0.885) were significant predictors for GSU (Table 4). However, core length was not predictive for GSU in multivariable analysis (*P* > 0.05) (Table 4). Multivariate analysis of patients with low-risk PCa who met the criteria for very low-risk PCa (clinical stage T1c, PSA <10 ng/ml, PSA density ≤0.15 ng/ml/gm, biopsy Gleason sum ≤6, 2 or fewer positive cores on biopsy, 50% or less involvement of any core with cancer, and 12 or fewer cores sampled) [15] also showed that core length was not a significant prognostic factor for GSU.

**Table 4:** Univariate and multivariate analyses of clinicopathological factors predicting Gleason score upgrading (GSU) among men diagnosed with low-risk prostate cancer.

	Univariate		Multivariate	
	OR (95% CI)	*P* value	OR (95% CI)	*P* value
Age (years)	1.024 (0.990–1.059)	0.169	1.060 (1.019–1.102)	0.004
BMI (kg/m^2^)	0.993 (0.910–1.084)	0.876	1.040 (0.943–1.149)	0.431
PSA (ng/ml)	1.106 (0.979–1.250	0.105	1.160 (1.008–1.336)	0.038
Prostate volume (ml)	0.965 (0.949–0.982)	< 0.001	0.960 (0.941–0.981)	< 0.001
Number of biopsy cores taken	0.647 (0.483–0.866)	0.003	0.637 (0.458–0.885)	0.007
Core length (mm)	1.010 (0.881–1.157)	0.889	1.089 (0.909–1.305)	0.356
Percentage of positive cores (%)	1.049 (1.026–1.073)	< 0.001	1.033 (0.989–1.078)	0.141
Maximum percentage of tumor length in a positive core (%)	1.044 (1.024–1.064)	< 0.001	1.039 (0.997–1.083)	0.069
Percentage of total tumor length in cores (%)	1.284 (1.145–1.440)	< 0.001	0.901 (0.664–1.222)	0.502
Clinical stage (T2a vs T1c)	1.124 (0.630–2.005)	0.692	1.272 (0.667–2.428)	0.465

Abbreviations: OR, odds ratio; CI, confidence interval; BMI, body mass index; PSA, prostate-specific antigen

Interestingly, although a positive correlation was identified between core length and prostate volume in our entire cohort undergoing TRUS-guided biopsy (Pearson correlation test r = 0.057, *P* < 0.001), there was no significant correlation between core length and prostate volume in the cancer group and low-risk PCa group (*P* > 0.05). Further, no significant correlation between mean core length obtained by PBx and the length of PCa tissue in the PBx specimen was found (*P* > 0.05).

## Discussion

To our knowledge, the present study is the first to evaluate the clinical value of core length in prostate biopsy for the prediction of GSU in patients with low-risk PCa. Previous studies demonstrate that the DR for PCa correlates with biopsy length, which is also related to the quality of the prostate biopsy [16–18]. Our series consisted of the largest cohort among the few studies that have focused on core length in PBx.

The importance of number and location of PBx cores remains controversial. Many studies on number and location of cores have been reported, but there is little data about the clinical value of core length. The core length in PBx can be influenced by characteristics of the biopsy needle, the technique of the doctor who is performing the biopsy, the site from which the specimen is sampled, and the method through which the biopsy tissues are collected, such as pre-embedding [16, 17, 19]. In general, a longer biopsy core length may be beneficial in a cancer group and a non-GSU group. This is because obtaining a longer biopsy core may improve histopathological sampling and overall sampling of cancers, especially high-grade cancers that are located deeper within the prostate gland. Suboptimal or inadequate core length during PBx increases the risk of missing PCa detection, because shorter biopsy cores reduce the quantity of tissue available for histopathological diagnosis [6, 16–18, 20]. Few studies have reported that PCa detection is influenced by core length in random PBx [7, 17, 18]. Iczkowski *et al*. found a positive correlation between core length and PCa detection in patients receiving sextant PBx. However, this correlation was only significant for the prostate apex, which is where the core length is shorter (11.8 mm) than that of the prostate base or mid-gland [17]. Obek *et al*. reported that the mean core length of a PCa group (12.3 mm) was longer than that of the non-cancer group (11.4 mm); they suggested that a cut-off length of 11.9 mm for PCa detection in multicore PBx [18]. However, Ficarra *et al*. reported that the length of cores diagnosed with PCa was similar to those diagnosed with non-cancer as long as the length of cores satisfied the criteria for standard quality in transperineal PBx [21]. The mean length of needle cores sampled during TRUS-guided PBx in our series surpassed the best quantitative standards of the optimal biopsy length recommended in past studies [6, 18, 20]. Our analysis of the full cohort of patients who received TRUS-guided PBx also showed that the mean length of patients diagnosed with a benign pathology (16.1 ± 1.9 mm) was longer than the recommended standards and similar to that of patients with a diagnosis of PCa (16.1 ± 1.8 mm). Our results could be explained by a hypothesis consistent with the concept of saturation in physical chemistry. The PCa DR did not increase with longer sampling of prostate tissue if the core length of PBx approached a plateau for PCa diagnosis.

This issue of core length is similar to that of the number of biopsy cores and needle size. Increasing the number of biopsy cores or the use of a higher caliber needle may improve histopathological sampling and ultimately improve the accuracy of diagnosing PCa. Jiang *et al*. reported that an initial saturation PBx scheme is more efficient than an extended PBx scheme in terms of PCa detection, especially for men with lower PSA, larger prostate volume, or lower PSA density [5]. However, a meta-analysis performed by Eichler *et al*. showed that taking more than 12 cores during an initial PBx added no significant benefit if the sample sites were as far lateral and posterior as possible in the peripheral gland [3]. According to the literature and the major international guidelines, the 12 cores extended scheme remains the recommended standard in the initial PBx setting [1, 3]. Despite the aforementioned assumption about needle size, it has also been reported that needle size does not affect the PCa DR and sample quality [22, 23]. Therefore, additional sampling does not guarantee increased diagnosis of PCa, although sampling an adequate amount of prostate tissue beyond the standard is essential for exact pathological diagnosis.

A shorter biopsy tissue length might be expected in the GSU group with smaller prostates. Interestingly, our results showed no significant correlation between core length and prostate volume in the cancer group and low-risk PCa group (*P* > 0.05). Thus, our results that the GSU group with smaller prostate volume demonstrated a similar biopsy core length as that of the non-GSU group with larger prostate volume might be reasonable. Core length was not a significant predictor of GSU after adjusting for the effect of prostate volume in multivariate analysis. In other words, obtaining a longer core may not be associated with improved prediction of GSU even if sufficient tissue was sampled during TRUS-guided PBx. There have been no published reports showing that sampling longer biopsy cores is associated with reduced likelihood of GSU.

The limitations of our study include its retrospective nature, the small variance of core length between groups, and data from a single institution. We could not assess which part of the prostate, including cores with a diagnosis of cancer during TRUS-guided PBx, was upgraded to high-grade PCa in radical prostatectomy specimens. The prognostic value of GSU in our series was limited, because Gleason score is a powerful but imperfect predictor of cancer-specific survival.

## Conclusions

The core length may not have significant impact on PCa detection in patients receiving contemporary multi (≥12)-core PBx and also GSU following radical prostatectomy among low-risk PCa group if an optimal or adequate core length is obtained during PBx for accurate histopathological diagnosis. Our results may provide additional information for counseling men who are considering PBx for diagnosis of PCa or AS as an alternative to curative treatment after a diagnosis of low-risk PCa. A large-scale, multicenter, prospective study is needed to fully evaluate the clinical significance of core length in PBx.

## Supporting Information

S1 Table The clinicopathological data in the present study. (XLSX)
